# Neutrophil depletion for early allogeneic islet survival in a methacrylic acid (MAA) copolymer-induced, vascularized subcutaneous space

**DOI:** 10.3389/frtra.2023.1244093

**Published:** 2023-10-27

**Authors:** So-Yoon Won, Sean M. Kinney, Michael V. Sefton

**Affiliations:** ^1^ Institute of Biomedical Engineering, University of Toronto, Toronto, ON, Canada; ^2^ Department of Chemical Engineering and Applied Chemistry, University of Toronto, Toronto, ON, Canada

**Keywords:** islet transplantation, immunosuppression, methacrylic acid, prevascularization, neutrophil depletion.

## Abstract

Islet transplantation is a promising treatment for type I diabetes (T1D). Despite the high loss of islets during transplantation, current islet transplant protocols continue to rely on portal vein infusion and intrahepatic engraftment. Because of the risk of portal vein thrombosis and the loss of islets to instant blood mediated inflammatory reaction (IBMIR), other transplantation sites like the subcutaneous space have been pursued for its large transplant volume, accessibility, and amenability for retrieval. To overcome the minimal vasculature of the subcutaneous space, prevascularization approaches or vascularizing biomaterials have been used to subcutaneously deliver islets into diabetic mice to return them to normoglycemia. Previous vascularization methods have relied on a 4 to 6 week prevascularization timeframe. Here we show that a vascularizing MAA-coated silicone tube can generate sufficient vasculature in 2 to 3 weeks to support a therapeutic dose of islets in mice. In order to fully harness the potential of this prevascularized site, we characterize the unique, subcutaneous immune response to allogeneic islets in the first 7 days following transplantation, a critical stage in successful engraftment. We identify neutrophils as a specific cellular target, a previously overlooked cell in the context of subcutaneous allogeneic islet transplantation. By perioperatively depleting neutrophils, we show that neutrophils are a key, innate immune cell target for successful early engraftment of allogeneic islets in a prevascularized subcutaneous site.

## 1. Introduction

Cell transplantation is a promising therapy for currently incurable diseases like type I diabetes (T1D) (1). Islet transplantation offers patients an opportunity to live without daily insulin injections by replacing islets that have been lost through autoimmune-mediated destruction (2). Despite the high loss of islets during portal vein infusion, current islet transplantation protocols continue to rely on intrahepatic engraftment (3). Because of the loss of islets to the immediate blood mediated inflammatory response (IBMIR) and the risk of portal vein thrombosis, other transplant sites that can support islets have been investigated (4–7). The subcutaneous site offers a large transplant volume, retrievability, and is a less invasive alternative to other sites such as the liver, spleen, and kidney capsule (5). As unique as are the opportunities of this site, it presents unique challenges. The subcutaneous site is not well-vascularized, but by using a prevascularization approach or vascularizing biomaterials, islets can be transplanted subcutaneously to return mice to normoglycemia (8–11). A methacrylic acid (MAA)-co-isodecyl acrylate (IDA) coated silicone tube has been shown to sufficiently vascularize the space to support islets in mice without the addition of growth factors or cells and to vascularize the tissue in 2 to 3 weeks, a shorter period than the 4 to 6 weeks required by other methods (8, 9). For translatability, the shorter time frame will allow for earlier islet transplantation.

The subcutaneous site also presents the challenge of its swift and robust immune response to foreign antigens. Because the skin is the first barrier of defense against invading pathogens, dermis-resident immune cells are prepared to respond to insult and can readily migrate to the adjacent layer of subcutaneous tissue (12). As one example, the effectiveness of the subcutaneous immune response has been studied and used to drive immune responses in the context of vaccinations (13). The subcutaneous route of vaccination leverages the presence of tissue-resident and migratory macrophages and dendritic cells to drive immunogenicity (14). However, for cell transplantation, this immune response presents a challenge for successful engraftment of allogeneic cells. Although subcutaneous immune responses have been well-characterized in non-vascularized tissue in response to antigen delivery through vaccination or infection (12, 13), prevascularization may alter the immune response to allogeneic antigens. This may be via MAA's ability to polarize macrophages (15, 16) and/or the effect of chemokines and chemotaxic signals binding to glycosaminoglycans on the new blood vessel's endothelial cells (ECs) (17, 18) which may not be specific to MAA and also relevant to other prevascularization methods. Our short-term, MAA-coated silicone tube prevascularization approach was used to study the vascularized, subcutaneous immune response to allogeneic islets. We identified acute neutrophil recruitment as a critical point in transplant rejection. Through perioperative depletion of neutrophils, we show that these innate cells are a key target for successful, subcutaneous engraftment of allogeneic islets.

## 2. Materials and methods

### 2.1. Animals

All animal procedures and protocols were approved by the University of Toronto Animal Care Committee. Animals were housed under sterile conditions at the University of Toronto's Division of Comparative Medicine (DCM). Islet transplant recipient BALB/c mice and islet donor C57BL/6J were procured from Charles River and Jackson Laboratories respectively.

### 2.2. Islet isolation

Islets were harvested from 7 to 9 weeks old C57BL/6 mice. The isolation modifies a previously described protocol (57). In brief, the pancreases were cannulated and perfused with collagenase (CIzyme RI, VitaCyte), then digested at 37°C. The islets were separated from the pancreatic debris by density gradient (Histopaque-1077 and Histopaque-1119, Sigma) followed by hand-picking. Primary mouse islets were cultured in islet medium (RPMI-1640 medium (Gibco) supplemented with 10% fetal calf serum (FCS) (Gibco), and 1% penicillin-streptomycin (Gibco)). Islet equivalent units (IEQ) were calculated based on the volumetric assumption of 150 *μ*m diameter of an islet.

### 2.3. Allogeneic subcutaneous response characterization

Silicone tubes (3 cm long) were dip-coated in a MAA-co-IDA solution (40% mol in THF), gas-sterilized, then inserted into a bluntly dissected tunnel on the upper dorsum of BALB/c mice. Uncoated silicone tubes were used as a control. After 14 days, 250 IEQ, which were isolated from C57BL/6J mice, were suspended in 20 *μ*l of neutralized PureCol Type I Collagen Solution (Advanced BioMatrix). The mixture was drawn into PE90 tubing and gelled at 37°C for 30 min–1 h. To access the transplant site, an incision was made close to the upper dorsum of the prevascularized BALB/c mice. The tube was flushed with PBS, and the tubing containing the islets (in collagen) was inserted into the larger tube. The larger tube acted as a guide to inject islets into the prevascularized site. Both tubes were removed upon injection, and the incision was sutured. Surgeries were done on anesthetized mice using inhaled isoflurane (induction at 3%–5%, maintenance at 2%–3%; provided by University of Toronto's DCM). All mice received ketoprofen (10 mg/kg; provided by University of Toronto's DCM) at the time of surgery, one day post-surgery, and up to 3 days post as required. To analyze the immune response by flow cytometry, islet grafts were excised and digested using the Tumor Dissociation Kit (Miltenyi Biotec Inc.) according to manufacturer's instructions. The single-cell suspension was then stained and analyzed by flow cytometry.

### 2.4. Flow cytometric analysis of subcutaneous tissue

Digested subcutaneous tissue (as outlined above) was stained with flow cytometry antibodies in in FACS Buffer (PBS with 0.5% BSA and 1 mM EDTA) in the dark and on ice, using two different flow cytometry panels, one specific for innate cells (CD3-BV605, CD11b-BV510, CD11c-PE-Cy5, CD45-BV650, CD206-PE-Cy7, F4/80-APCe780*, Ly6C-AF700, Ly6G-PE-Dazzle, MHCII-APC, and Viability-Fixable Blue*) and the other for adaptive and natural killer (NK) cells (CD3-BV605, CD4-FITC, CD8-e506, CD25-BV421, CD44-PE-Cy5, CD45-BV650, CD49b-PE/Dazzle, CD62l-PE, CD69-PE-Cy7, FoxP3-AF647, Viability-Fixable Blue*). Antibodies were purchased from Biolegend or *Invitrogen. Precision Count Beads (Biolegend) were added to the samples before collection. Samples were acquired on the BD X-20, and data analyzed using FlowJo software.

### 2.5. Allogeneic islet transplantation in prevascularized site

Prevascularized BALB/c mice (as outlined above) were made diabetic by a single intraperitoneal injection of streptozotocin (180 mg/kg, Sigma) one week before transplantation. Mice were considered diabetic if they had blood glucose levels above 20 mM for at least two consecutive days. After 14–21 days of prevascularization, 600 IEQ which were isolated from C57BL/6J mice, were suspended in 20 μl of neutralized PureCol Type I Collagen Solution (Advanced BioMatrix). The mixture was drawn into PE90 tubing and gelled at 37°C for 1 h. The islets were delivered to the prevascularized site as outlined above.

Daily blood glucose measurements were done via tail vein and measured with a glucometer (OneTouch Ultra 2, Lifescan). Insulin (25 units/kg Humulin *N*; 25 units/kg Humulin *R*; Lilly) was administered when blood glucose levels were greater than 25 mM.

Mice were immunosuppressed with a single or a combination of drugs perioperatively. Mice received dexamethasone (5 mg/kg, Sigma), fingolimod (1 mg/kg, Sigma), rapamycin (0.2 mg/kg, Sigma), or InVivoPlus anti-mouse Ly6G (40 μg/mouse, Bio × Cell) with daily intraperitoneal injections as outlined in the figures.

### 2.6. Flow cytometry of peripheral immune cells

Peripheral blood immune cells were monitored by taking peripheral blood via tail vein. Blood was collected into red blood cell (RBC) lysis buffer [0.15 M ammonium chloride, 10 mM potassium bicarbonate, 0.1 mM ethylenediaminetetraacetic acid (EDTA)], and left to shake at room temperature for 30 min. The solution was centrifuged, washed, and the cells were stained for immune markers: Ly6G-PE/Dazzle, CD4-FITC, CD8-e506*, CD25-BV421, FoxP3-AF647 (Biolegend or *Invitrogen). Samples were acquired on the BD X-20, and data analyzed using FlowJo software.

### 2.7. Statistics

Statistical analysis was performed using GraphPad Prism (Version 8). Statistical comparison between groups was performed using one- or two-way ANOVA with a Tukey multiple comparisons test, or an unpaired two-tailed *t*-test, as appropriate. Comparisons were considered significant with *p* < 0.05. Data presented are mean ± SEM. Each ‘*n*’ represents a biological replicate.

## 3. Results

### 3.1. Acute neutrophil recruitment occurs post-transplantation

**Figure 1:**
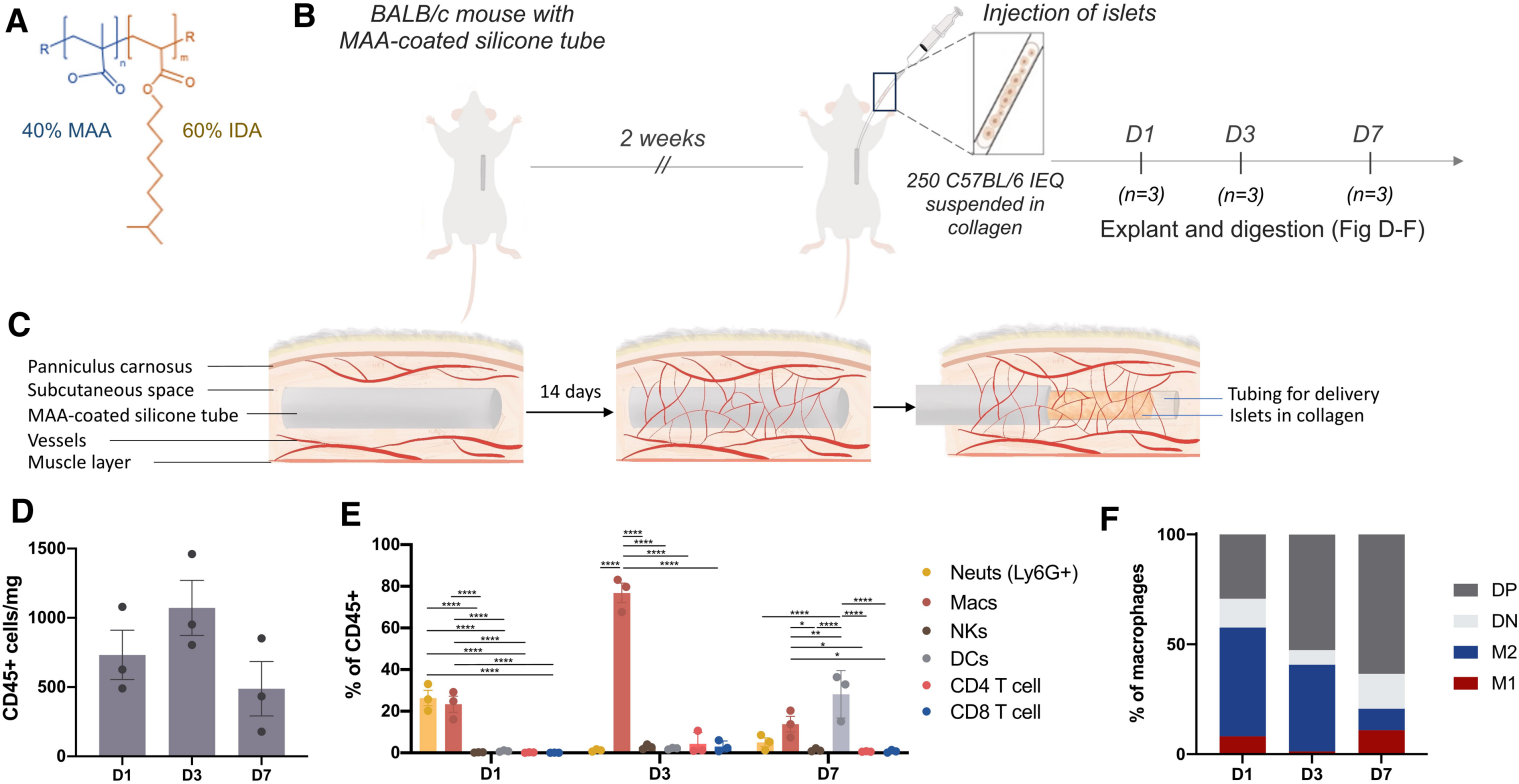
Neutrophils were the predominant immune cell in the first 24 h post-islet transplantation. Silicone tubes (3 cm long) were dip-coated in a 40% MAA-co isodecyl acrylate (IDA) solution (**A**), then inserted into the upper dorsum of BALB/c mice. After 14 days, 250 islet equivalents (IEQ) isolated from C57Bl/6J mice were suspended in collagen and injected into the subcutaneous site at the time of silicone tube removal (**B,C**). The subcutaneous tissue was digested and the immune response to the 250 allogeneic islets was analyzed by flow cytometry 1, 3, and 7 days after transplantation (**D–F**). (**D**) There was the greatest CD45+ immune cell recruitment 3 days post transplantation. (**E**) Live, single, CD45+ immune cells were further gated to reveal a dynamic wave of neutrophil then macrophage and then dendritic cell (DC) recruitment in the week following transplantation. Neutrophils were gated as F480-Ly6G+; macrophages as F480+CD11b+; natural killer cells (NKs) as CD49b+; DCs as F480-CD11c+. (**F**) Macrophage polarization was further determined by CD206 and MHCII expression. M1(CD206-MHCII+); M2(CD206+MHCII-); DP (CD206+MHCII+); DN(CD206-MHCII-). Data shown as mean ± SEM; *n* = 3-6; analyzed using two-way ANOVA; *p < 0.05, **p < 0.005, ****p < 0.0001.

To better understand the subcutaneous immune response following vascularization with an MAA-coated silicone tube, allogeneic islets [250 islet equivalents (IEQ), C57/BL6] were suspended in collagen then delivered into the prevascularized site as outlined above (Figures 1A–C); this dose of islets is subtherapeutic and animals were not diabetic. Digestion and flow cytometric analysis of the tissue at various timepoints over the first week post-transplantation revealed waves of different immune cell recruitment to the site with the greatest number of immune cells recruited 3 days post-transplantation (Figure 1D). Within the first 24 h post-transplantation, neutrophils and macrophages were the prominent cell populations. By day 3, macrophages were the majority of the CD45 + immune cells at the site whereas the neutrophil response had subsided. By day 7, macrophages were no longer the predominant immune cells, and dendritic cells (DCs), the professional antigen presenting cells (APCs) which go on to deliver priming and activating signals to T cells, were at the site (Figure 1E). Classical macrophage polarization markers (CD206, MHCII) revealed an increase in double-positive macrophages, and a trend for decreased M2 macrophages (CD206 + MHCII-) over the course of 7 days (Figure 1F).

### 3.2. Neutrophils are responsible for early graft failure

**Figure 2:**
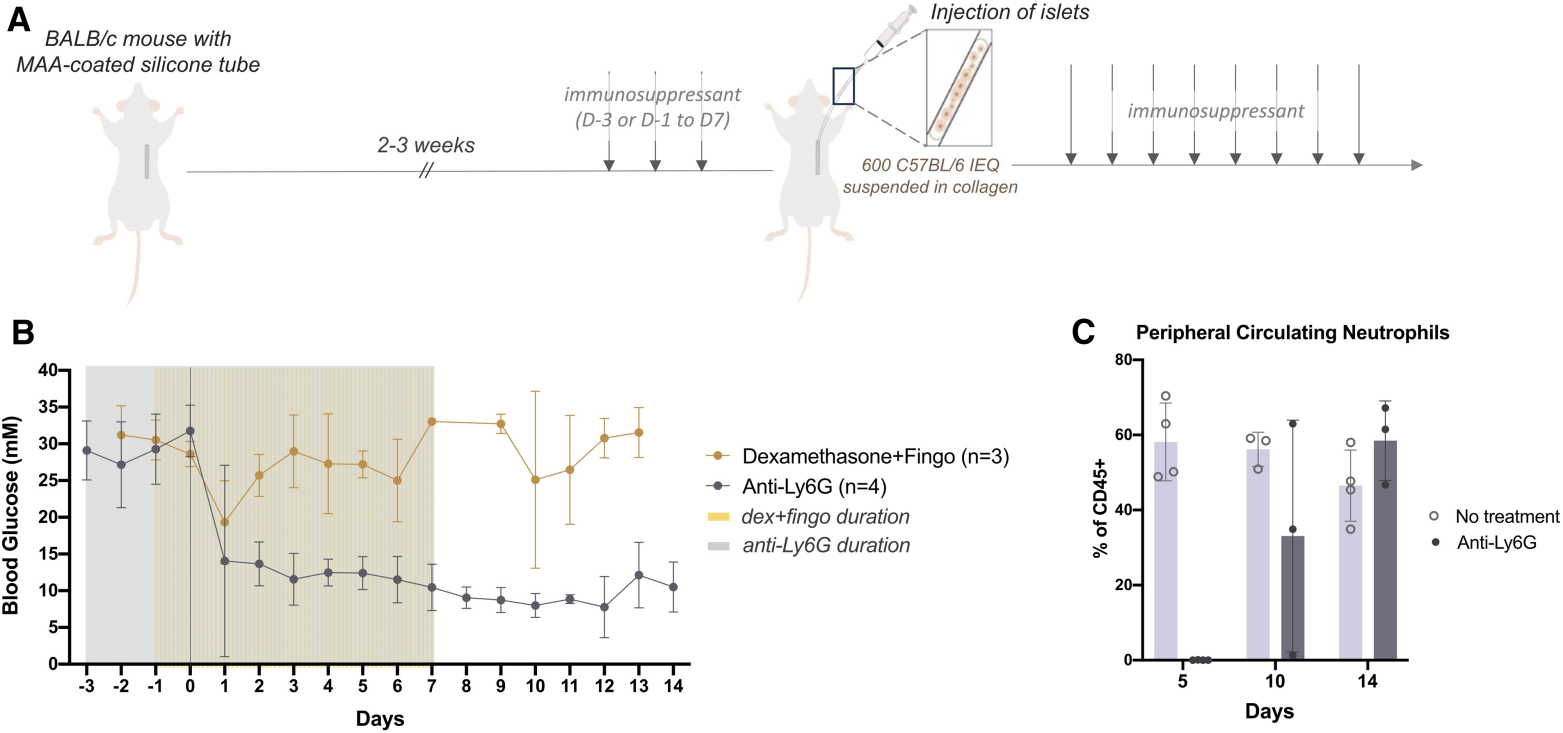
Perioperative anti-Ly6G treatment returned diabetic mice to normoglycemia post-transplantation even after neutrophils began to return (**A**) after 14-21 days of prevascularization, 600 islet equivalents (IEQ) isolated from C57Bl/6J mice were suspended in collagen and injected into the subcutaneous site of diabetic BALB/c mice at the time of silicone tube removal. Mice received daily immunosuppressants, dexamethasone (5 mg/kg, i.p.) and fingolimod (1 mg/kg, i.p.) from 1 day before (D-1) to 7 days after (D7) transplantation, or anti-Ly6G alone (40 μg/mouse, i.p.) from 3 days before (D-3) to 7 days after (D7) transplantation. (**B**) Blood glucose (BG) levels of diabetic BALB/c mice receiving prednisolone and fingolimod (*n* = 3) remained high while the BG of mice receiving only anti-Ly6G (*n* = 3) all dropped to normoglycemic range after transplantation. (**C**) The proportion of CD45+ Ly6G+ cells in peripheral blood was examined by flow cytometry to monitor the effect of the anti-Ly6G antibody during (D5) and after stopping treatment (D10, D14). The effect of the antibody was compared to a no treatment control. Data shown as mean ± SEM; *n* = 3–4.

Because macrophages and neutrophils were the predominant immune cell populations recruited at the early time points, we sought to target this inflammation. BALB/c mice which had been rendered diabetic using streptozotocin were given immunosuppressants perioperatively to broadly or specifically target the recruited innate cells (Figure 2A). Dexamethasone, a steroid that dampens inflammation (17, 18) and fingolimod, an immunosuppressant that prevents immune cell egress from lymph nodes by binding to sphingosine-1-phosphate receptors (S1PR) (19), or an anti-Ly6G neutralizing antibody (anti-Ly6G) that specifically targets neutrophils (20) were administered intraperitoneally and perioperatively as outlined (Figure 2A). 3–5 days after the mice were diabetic and 3 days after the first drug dose, allogeneic islets (600 IEQ, C57BL/6J) were delivered to the prevascularized site. Even when dexamethasone was administered in conjunction with fingolimod, the grafts failed within 2 days. However, the anti-Ly6G alone was sufficient for successful early (14 days) islet engraftment (Figure 2B). These results suggest that it is the neutrophils that were responsible for early graft failure.

To confirm the effect of the anti-Ly6G antibody, peripheral blood was taken from the mice during, and 3 and 7 days after stopping the treatment; anti-Ly6G treatment had begun 3 days before islet transplant and continued for 7 days post-transplant. During the treatment, there were no circulating neutrophils. 3 days after stopping treatment or 10 days post-transplantation (D10), we observed neutrophils returning in some mice (Figure 2C). By 7 days after stopping treatment (D14), neutrophil populations returned, but these mice remained normoglycemic (Figures 2B,C). Therefore, neutrophils played a critical role in preventing islet engraftment at early timepoints, but they were not the key cellular target after these early days.

### 3.3. Adaptive immune response is dampened with short-term, low-dose rapamycin

**Figure 3:**
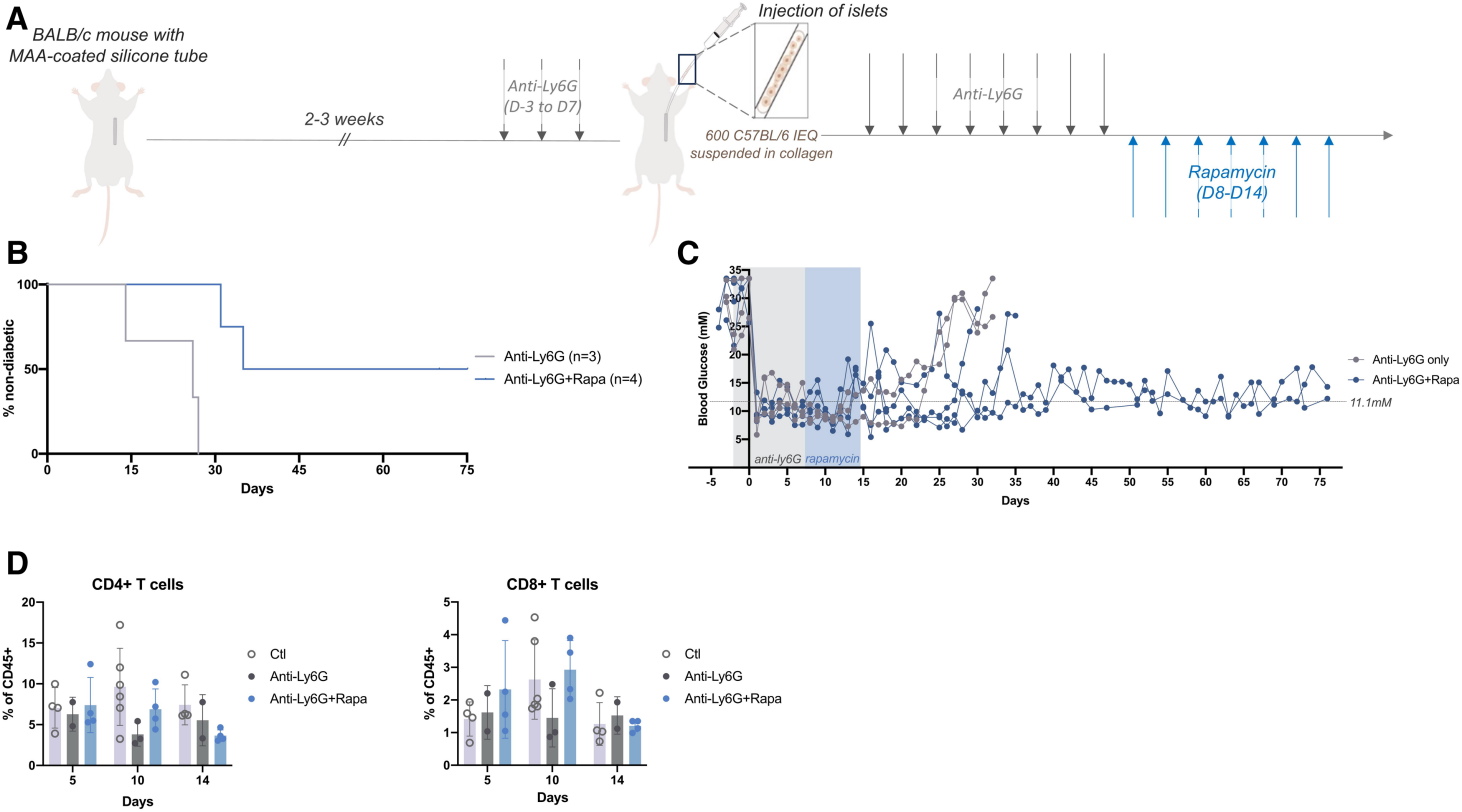
Low dose rapamycin enabled longer term blood glucose control and but did not alter systemic T cells proportions. (**A**) After 14-21 days of prevascularization, 600 islet equivalents (IEQ) isolated from C57Bl/6J mice were suspended in collagen and injected into the subcutaneous site of diabetic BALB/c mice at the time of silicone tube removal. All mice received daily anti-Ly6G (40 μg/mouse; i.p.) from 3 days before (D-3) to 7 days after (D7) transplantation, and some mice continued to receive an immunosuppressant (rapamycin, 0.2 mg/kg, i.p.) for another 7 days (D8-D14). (**B,C**) Grafts failed earlier in mice receiving only anti-Ly6G (*n* = 3) whereas mice that received subsequent rapamycin remained normoglycemic for >30 days with some grafts still surviving past 75 days post-transplantation (*n* = 4). Mice were considered diabetic when they had BG levels above 20 mM for two consecutive readings. (**D**) Peripheral blood samples showed little changes in peripheral T cell (CD45+ Ly6G- CD4+; CD45+ Ly6G- CD8+) populations after 7 daily doses of rapamycin (D14). Data shown as mean ± SEM; *n* = 2-5.

Because T cells are responsible for driving graft rejection in the weeks following transplantation, low dose rapamycin was administered for 7 days following anti-Ly6G treatment (Figure 3A). Rapamycin targets mTOR (mammalian target of rapamycin), a key signaling molecule in T cells for effective response to antigen recognition and differentiation into effector T cells (21, 22). Rapamycin inhibits this activation and helps to bias the T cells into a regulatory phenotype (23–25). Despite a short term and low dose of rapamycin, mice that received rapamycin remained normoglycemic for several weeks after the end of the treatment (Figures 3B,C). Evaluation of the peripheral immune cells revealed that the anti-Ly6G plus rapamycin treatment slightly decreased the peripheral CD4 + and CD8+ T cell populations by the end of the 7 days treatment (D14) as compared to T cell percentages in this treatment group at earlier timepoints (Figure 3D); however these differences were not significant. Peripheral T cells may not capture the effectiveness of such short-term, low-dose rapamycin after anti-Ly6G treatment; yet longer term rapamycin may help to further prolong graft survival time.

## 4. Discussion

To fully harness the potential of the subcutaneous space as a cell transplantation site, better understanding and fine-tuned targeting of the subcutaneous immune response is required. Without immunosuppression, allogeneic islet transplants failed within the first 48 h post-transplantation. Even with the administration of rapamycin, a widely used immunosuppressant in transplantation, perioperative rapamycin alone was unable to return mice to normoglycemia in the early days post-transplantation (data not shown). Therefore, we aimed to identify and target an earlier, innate immune cell to prevent this early islet rejection. Characterization of the vascularized, subcutaneous transplant microenvironment revealed macrophage and neutrophil recruitment in the early days following transplantation which we speculated to be responsible for the early failure of the grafts. The early inflammation was targeted broadly using a steroid and fingolimod or specifically with a neutrophil targeting anti-Ly6G antibody. Dexamethasone, even co-administered with fingolimod, was unable to dampen the immune response sufficiently for islet survival. This combination was administered starting only one day before transplantation because immunosuppression in the context of islet transplantation has moved away from steroids because of their effects on metabolism and resulting glucotoxicity (26).

Surprisingly, the anti-Ly6G antibody alone however, was able to return all mice to normoglycemia in the early days post-transplantation through specific depletion of neutrophils. Neutrophils are bone marrow-derived, post-mitotic cells and only survive for a few days in circulation (27). Because neutrophils also extravasate to tissue in response to inflammation, tissue damage, and chemokines like CXCL1/2/3, and CXCL8 secreted by activated macrophages (28, 29) and DCs (30–32), anti-Ly6G was administered up to 7 days after transplantation while the innate immune response was resolved. However, it is plausible that an even shorter depletion period is sufficient for islet survival.

Because of their short lifespan, it was expected that neutrophils would begin to return after stopping anti-Ly6G treatment, as we observed. Despite the return in neutrophils, partially by 3 days and completely by 7 days after stopping treatment, the mice remained normoglycemic. This suggested the key role of neutrophils in the early days post-transplantation but that they are not the main mediator of rejection after this critical period. Although neutrophils may not directly play a role at this time point, the lack of signals provided by the neutrophils and therefore the missing neutrophil-triggered immune response may have a long-lasting effect in modulating the subsequent response. Preliminary immunofluorescent staining (data not shown) of an iDISCO cleared explant at day 75 suggested that some islets (insulin+) were not in fact isolated from immune cell (CD45+) contact but rather survived despite their presence, perhaps due to long-lasting immunomodulation by the early neutrophil depletion and subsequent short-term rapamycin. CD31 + staining likely from endothelial cells was also seen throughout the explant.

Neutrophils relay information to other immune cells to shape and influence their responses (33–37). Although neutrophils may mediate the islet rejection themselves by releasing cytokines, reactive oxygen species (ROS), and neutrophil extracellular traps (NETs) as they extravasate en masse to the transplant site, there is increasing appreciation for the role of neutrophils in determining the subsequent immune response (35, 38, 39). Neutrophils and NETs increase antigen presentation, dendritic cell (DC) recruitment and macrophage cytokine production (36, 37, 40); neutrophils also affect B and T cell activation (35, 37, 41, 42). Therefore, eliminating the inflammatory first impression that neutrophils signal— dampening antigen presentation and cytokine production by other immune cells, allows the ensuing immune response to be manageable even with short-term and low-dose rapamycin as we observed. The short-term rapamycin may be further aiding the lack of neutrophil-driven responses to generate a locally, immune privileged site around the islets maintained by regulatory T cells (23, 24) and endothelial cells (25)—effects that would not be captured peripherally. Although longer term or slow-release rapamycin (43) was not explored in this study, it could help to further prolong graft survival even at a low dose.

Although neutrophils have previously been considered in the context of islet transplantation outside the subcutaneous space, inhibition of neutrophil infiltration has shown varying efficacy in the liver and under the kidney capsule (44, 45). Neutrophil infiltration was inhibited using reparixin which blocks the CXCR1/2 axis, chemotaxic receptors which are found on neutrophils, natural killer T cells (46), and macrophages (47). It showed no increased benefit as compared to cytotoxic T lymphocyte-associated antigen-4-Ig (CTLA4-Ig) monotherapy (44), but there was a benefit of reparixin alone as compared to a vehicle in hepatic engraftment and the combined effect of reparixin with MMF and FK506 with and without CTLA4-Ig prolonged graft survival up to approximately 30 days but not longer term survival (45). Because reparixin does not solely target neutrophils, graft rejection cannot be attributed to only neutrophils in these contexts. A reparixin supplemented immunosuppression protocol in human clinical trials showed 2 of 4 patients achieving insulin independence after a second intrahepatic islet transplantation (NCT01220856). Intrahepatic transplantation exposes islets to IBMIR which may not be sufficiently managed with reparixin, and it may show greater benefit with islet transplantation in a different transplantation site like the subcutaneous space. Because complete neutrophil depletion is a preliminary approach to address the role of neutrophils in subcutaneous islet transplantation, it is a potential avenue of study to evaluate the benefit of reparixin, an already clinical trial approved drug, in our subcutaneous system by preventing neutrophil and NKT infiltration to evaluate if specific and complete depletion is required.

By solving the poor vascularization caveat of the subcutaneous space with a quickly vascularizing MAA-coated tube, the tissue became better surveilled with more vasculature bringing in more neutrophils. However, specific depletion of these cells allowed for early islet engraftment and longer-term survival with short, low-dose rapamycin to manage the adaptive T cell response. It is a limitation of this study that the exact mechanism by which neutrophil depletion leads to islet survival remains unclear. Although we have not elucidated whether it is the neutrophil's role as “executioner” or “messenger” that is critical to manage for graft survival, this study focuses attention to neutrophils, a cell previously disregarded in subcutaneous islet transplantation. As the field of oncology and the pharmaceutical industry begin to better appreciate the role of neutrophils and design neutrophil-targeting checkpoint inhibitors (48), we provide evidence that there is an unappreciated role of neutrophils that remains to be leveraged at least perioperatively, in the context of allogeneic islet transplantation as well. Through better understanding of neutrophils’ consequential effects, it may become possible to elegantly tune the immune response through this cell to help unlock the full potential of the subcutaneous space as a transplant site.

## Data Availability

The raw data supporting the conclusions of this article will be made available by the authors, without undue reservation.

## References

[1] Shapiro AMJ Pokrywczynska M Ricordi C. Clinical pancreatic islet transplantation. Nat Rev Endocrinol. (2017) 13(5):268–77. 10.1038/nrendo.2016.178 27834384

[2] DiMeglio LA Evans-Molina C Oram RA. Type 1 diabetes. Lancet. (2018) 391(10138):2449–62. 10.1016/S0140-6736(18)31320-5 29916386 PMC6661119

[3] Shapiro AMJ. Strategies toward single-donor islets of langerhans transplantation. Curr Opin Organ Transplant. (2011) 16(6):627–31. 10.1097/MOT.0b013e32834cfb84 22068022 PMC3268080

[4] Bennet W Groth CG Larsson R Nilsson B Korsgren O. Isolated human islets trigger an instant blood mediated inflammatory reaction: implications for intraportal islet transplantation as a treatment for patients with type 1 diabetes. Ups J Med Sci. (2000) 105(2):125–33. 10.1517/03009734000000059 11095109

[5] Pellegrini S. Alternative transplantation sites for islet transplantation. In: Orlando G Piemonti L Ricordi C Stratta RJ Gruessner RW, editors. Transplantation, bioengineering, and regeneration of the endocrine pancreas. Cambridge, USA: Elsevier (2020). p. 833–47.

[6] Sakata N Aoki T Yoshimatsu G Tsuchiya H Hata T Katayose Y Strategy for clinical setting in intramuscular and subcutaneous islet transplantation. Diabetes Metab Res Rev. (2014) 30(1):1–10. 10.1002/dmrr.2463 24000195

[7] Pepper AR Gala-Lopez B Ziff O Shapiro AMJ. Revascularization of transplanted pancreatic islets and role of the transplantation site. Clin Dev Immunol. (2013) 2013:1–13. 10.1155/2013/352315 PMC3782812 24106517

[8] Pepper AR Gala-Lopez B Pawlick R Merani S Kin T Shapiro AMJ. A prevascularized subcutaneous device-less site for islet and cellular transplantation. Nat Biotechnol. (2015) 33(5):518–23. 10.1038/nbt.3211 25893782

[9] Vlahos AE Talior-Volodarsky I Kinney SM Sefton M V. A scalable device-less biomaterial approach for subcutaneous islet transplantation. Biomaterials. (2021) 269:120499. 10.1016/j.biomaterials.2020.120499 33168223

[10] Coindre VF Carleton MM Sefton M V. Methacrylic acid copolymer coating enhances constructive remodeling of polypropylene mesh by increasing the vascular response. Adv Healthc Mater. (2019) 8(18):1900667. 10.1002/adhm.201900667 31407481

[11] Kinney SM Ortaleza K Vlahos AE Sefton M V. Degradable methacrylic acid-based synthetic hydrogel for subcutaneous islet transplantation. Biomaterials. (2022) 281:121342. 10.1016/j.biomaterials.2021.121342 34995903

[12] Richmond JM Harris JE. Immunology and skin in health and disease. Cold Spring Harb Perspect Med. (2014) 4(12):a015339–a015339. 10.1101/cshperspect.a015339 25452424 PMC4292093

[13] Cook IF. Evidence based route of administration of vaccines. Hum Vaccin. (2008) 4(1):67–73. 10.4161/hv.4.1.4747 17881890

[14] Turner MR Balu-Iyer S V. Challenges and opportunities for the subcutaneous delivery of therapeutic proteins. J Pharm Sci. (2018) 107(5):1247–60. 10.1016/j.xphs.2018.01.007 29336981 PMC5915922

[15] Lisovsky A Zhang DKY Sefton M V. Effect of methacrylic acid beads on the sonic hedgehog signaling pathway and macrophage polarization in a subcutaneous injection mouse model. Biomaterials. (2016) 98:203–14. 10.1016/j.biomaterials.2016.04.033 27264502

[16] Talior-Volodarsky I Mahou R Zhang D Sefton M. The role of insulin growth factor-1 on the vascular regenerative effect of MAA coated disks and macrophage-endothelial cell crosstalk. Biomaterials. (2017) 144:199–210. 10.1016/j.biomaterials.2017.08.019 28841464

[17] Rhen T Cidlowski JA. Antiinflammatory action of glucocorticoids — new mechanisms for old drugs. N Engl J Med. (2005) 353(16):1711–23. 10.1056/NEJMra050541 16236742

[18] Ramamoorthy S Cidlowski JA. Corticosteroids. Rheum Dis Clin N Am. (2016) 42(1):15–31. 10.1016/j.rdc.2015.08.002 PMC4662771 26611548

[19] Cohen JA Barkhof F Comi G Hartung HP Khatri BO Montalban X Oral fingolimod or intramuscular interferon for relapsing multiple sclerosis. N Engl J Med. (2010) 362(5):402–15. 10.1056/NEJMoa0907839 20089954

[20] Boivin G Faget J Ancey PB Gkasti A Mussard J Engblom C Durable and controlled depletion of neutrophils in mice. Nat Commun. (2020) 11(1):2762. 10.1038/s41467-020-16596-9 32488020 PMC7265525

[21] Mukherjee S Mukherjee U. A comprehensive review of immunosuppression used for liver transplantation. J Transplant. (2009) 2009:701464. 10.1155/2009/701464 20130772 PMC2809333

[22] Chi H. Regulation and function of mTOR signalling in T cell fate decisions. Nat Rev Immunol. (2012) 12(5):325–38. 10.1038/nri3198 22517423 PMC3417069

[23] Qu Y Zhang B Zhao L Liu G Ma H Rao E The effect of immunosuppressive drug rapamycin on regulatory CD4+CD25+Foxp3+T cells in mice. Transpl Immunol. (2007) 17(3):153–61. 10.1016/j.trim.2007.01.002 17331841

[24] Turnquist HR Raimondi G Zahorchak AF Fischer RT Wang Z Thomson AW. Rapamycin-conditioned dendritic cells are poor stimulators of allogeneic CD4+ T cells, but enrich for antigen-specific Foxp3+ T regulatory cells and promote organ transplant tolerance. The Journal of Immunology. (2007) 178(11):7018–31. 10.4049/jimmunol.178.11.7018 17513751

[25] Wang C Yi T Qin L Maldonado RA von Andrian UH Kulkarni S Rapamycin-treated human endothelial cells preferentially activate allogeneic regulatory T cells. J Clin Invest. (2013) 123(4):1677–93. 10.1172/JCI66204 23478407 PMC3613923

[26] Hwang JL Weiss RE. Steroid-induced diabetes: a clinical and molecular approach to understanding and treatment. Diabetes Metab Res Rev. (2014) 30(2):96–102. 10.1002/dmrr.2486 24123849 PMC4112077

[27] Lawrence SM Corriden R Nizet V. The ontogeny of a neutrophil: mechanisms of granulopoiesis and homeostasis. Microbiol Mol Biol Rev. (2018) 82(1):e00057–17. 10.1128/MMBR.00057-17 29436479 PMC5813886

[28] Knudsen E Iversen PO van Rooijen N Benestad HB. Macrophage-dependent regulation of neutrophil mobilization and chemotaxis during development of sterile peritonitis in the rat. Eur J Haematol. (2002) 69(5–6):284–96. 10.1034/j.1600-0609.2002.02657.x 12460233

[29] De Filippo K Henderson RB Laschinger M Hogg N. Neutrophil chemokines KC and macrophage-inflammatory protein-2 are newly synthesized by tissue macrophages using distinct TLR signaling pathways. The Journal of Immunology. (2008) 180(6):4308–15. 10.4049/jimmunol.180.6.4308 18322244

[30] Sallusto F Palermo B Lenig D Miettinen M Matikainen S Julkunen I Distinct patterns and kinetics of chemokine production regulate dendritic cell function. Eur J Immunol. (1999) 29(5):1617–25. 10.1002/(SICI)1521-4141(199905)29:05<1617::AID-IMMU1617>3.0.CO;2-3 10359116

[31] Scimone ML Lutzky VP Zittermann SI Maffia P Jancic C Buzzola F Migration of polymorphonuclear leucocytes is influenced by dendritic cells. Immunology. (2005) 114(3):375–85. 10.1111/j.1365-2567.2005.02104.x 15720439 PMC1782099

[32] GEIJTENBEEK T LUDWIGI Y VANKOOYK. Two way communication between neutrophils and dendritic cells. Curr Opin Pharmacol. (2006) 6(4):408–13. 10.1016/j.coph.2006.03.009 16750420

[33] Weber FC Németh T Csepregi JZ Dudeck A Roers A Ozsvári B Neutrophils are required for both the sensitization and elicitation phase of contact hypersensitivity. J Exp Med. (2015) 212(1):15–22. 10.1084/jem.20130062 25512469 PMC4291534

[34] Langereis JD Pickkers P de Kleijn S Gerretsen J de Jonge MI Kox M. Spleen-derived IFN-γ induces generation of PD-L1+-suppressive neutrophils during endotoxemia. J Leukoc Biol. (2017) 102(6):1401–9. 10.1189/jlb.3A0217-051RR 28974543

[35] Marini O Costa S Bevilacqua D Calzetti F Tamassia N Spina C Mature CD10^+^ and immature CD10^−^ neutrophils present in G-CSF–treated donors display opposite effects on T cells. Blood. (2017) 129(10):1343–56. 10.1182/blood-2016-04-713206 28053192

[36] Souwer Y Groot Kormelink T Taanman-Kueter EW Muller FJ van Capel TMM Varga D V Human TH17 cell development requires processing of dendritic cell–derived CXCL8 by neutrophil elastase. J Allergy Clin Immunol. (2018) 141(6):2286–2289.e5. 10.1016/j.jaci.2018.01.003 29391256

[37] Krishnamoorthy N Douda DN Brüggemann TR Ricklefs I Duvall MG Abdulnour REE Neutrophil cytoplasts induce T_H_17 differentiation and skew inflammation toward neutrophilia in severe asthma. Sci Immunol. (2018) 3(26):eaao4747. 10.1126/sciimmunol.aao4747 30076281 PMC6320225

[38] Mócsai A Abram CL Jakus Z Hu Y Lanier LL Lowell CA. Integrin signaling in neutrophils and macrophages uses adaptors containing immunoreceptor tyrosine-based activation motifs. Nat Immunol. (2006) 7(12):1326–33. 10.1038/ni1407 17086186 PMC4698344

[39] Burgener SS Schroder K. Neutrophil extracellular traps in host defense. Cold Spring Harb Perspect Biol. (2020) 12(7):a037028. 10.1101/cshperspect.a037028 31767647 PMC7328462

[40] Warnatsch A Ioannou M Wang Q Papayannopoulos V. Neutrophil extracellular traps license macrophages for cytokine production in atherosclerosis. Science (1979). (2015) 349(6245):316–20. 10.1126/science.aaa8064 PMC4854322 26185250

[41] Huard B McKee T Bosshard C Durual S Matthes T Myit S APRIL secreted by neutrophils binds to heparan sulfate proteoglycans to create plasma cell niches in human mucosa. J Clin Invest. (2008) 118(8):2887–95. 18618015 10.1172/JCI33760 PMC2447926

[42] Parsa R Lund H Georgoudaki AM Zhang XM Ortlieb Guerreiro-Cacais A Grommisch D BAFF-secreting neutrophils drive plasma cell responses during emergency granulopoiesis. J Exp Med. (2016) 213(8):1537–53. 10.1084/jem.20150577 27432941 PMC4986521

[43] Burke JA Zhang X Bobbala S Frey MA Bohorquez Fuentes C Freire Haddad H Subcutaneous nanotherapy repurposes the immunosuppressive mechanism of rapamycin to enhance allogeneic islet graft viability. Nat Nanotechnol. (2022) 17(3):319–30. 10.1038/s41565-021-01048-2 35039683 PMC8934301

[44] Pawlick RL Wink J Pepper AR Bruni A Abualhassen N Rafiei Y Reparixin, a CXCR1/2 inhibitor in islet allotransplantation. Islets. (2016) 8(5):115–24. 10.1080/19382014.2016.1199303 27328412 PMC5029202

[45] Citro A Cantarelli E Pellegrini S Dugnani E Piemonti L. Anti-Inflammatory strategies in intrahepatic islet transplantation: a comparative study in preclinical models. Transplantation. (2018) 102(2):240–8. 10.1097/TP.0000000000001925 28902069

[46] Citro A Cantarelli E Maffi P Nano R Melzi R Mercalli A CXCR1/2 Inhibition enhances pancreatic islet survival after transplantation. J Clin Invest. (2012) 122(10):3647–51. 10.1172/JCI63089 22996693 PMC3461913

[47] Bishayi B Adhikary R Sultana S Dey R Nandi A. Altered expression of CXCR1 (IL-8R) in macrophages utilizing cell surface TNFR1 and IL-1 receptor during Staphylococcus aureus infection. Microb Pathog. (2017) 113:460–71. 10.1016/j.micpath.2017.11.028 29162483

[48] Faget J Peters S Quantin X Meylan E Bonnefoy N. Neutrophils in the era of immune checkpoint blockade. J Immunother Cancer. (2021) 9(7):e002242. 10.1136/jitc-2020-002242 34301813 PMC8728357

